# Neural basis of adolescent THC-induced potentiation of opioid responses later in life

**DOI:** 10.1038/s41386-024-02033-8

**Published:** 2024-12-10

**Authors:** Elizabeth Hubbard, Pieter Derdeyn, Vivienne Mae Galinato, Andrew Wu, Katrina Bartas, Stephen V. Mahler, Kevin T. Beier

**Affiliations:** 1 Department of Physiology and Biophysics, University of California, Irvine, CA USA; 2 Program in Mathematical, Computational, and Systems Biology, University of California, Irvine, CA USA; 3 Department of Neurobiology and Behavior, University of California, Irvine, CA USA; 4 Department of Biomedical Engineering, University of California, Irvine, CA USA; 5 Department of Pharmaceutical Sciences, University of California, Irvine, CA USA

**Keywords:** Addiction, Motivation, Reward, Developmental disorders

## Abstract

Use of one addictive drug typically influences the behavioral response to other drugs, either administered at the same time or a subsequent time point. The nature of the drugs being used, as well as the timing and dosing, also influence how these drugs interact. Here, we tested the effects of adolescent THC exposure on the development of morphine-induced behavioral adaptations following repeated morphine exposure during adulthood. We found that adolescent THC administration paradoxically prevented the development of anxiety-related behaviors that emerge during a forced abstinence period following morphine administration but facilitated reinstatement of morphine CPP. Following forced abstinence, we then mapped the whole-brain response to a moderate dose of morphine and found that adolescent THC administration led to an overall increase in brain-wide neuronal activity and increased the functional connectivity between frontal cortical regions and the ventral tegmental area. Last, we show using rabies virus-based circuit mapping that adolescent THC exposure triggers a long-lasting elevation in connectivity from the frontal cortex regions onto ventral tegmental dopamine cells. Our study adds to the rich literature on the interaction between drugs, including THC and opioids, and provides potential neural substates by which adolescent THC exposure influences responses to morphine later in life.

## Introduction

Cannabis is currently the most widely used drug by adolescents in the United States [1]. Despite a reputation as a relatively harmless drug, over 30% of cannabis users meet the diagnostic criteria for cannabis use disorder [2], a similar proportion to those who become addicted after trying “hard” drugs such as cocaine and opioids [3]. With the global trend toward cannabis legalization enabling increased access to cannabis, it is critical to understand how adolescent cannabis use impacts the brain and behavior, especially if it does so in a persistent manner in the developing adolescent brain. Adolescent exposure to the major psychoactive constituent of cannabis, Δ-9-tetrahydrocannabinol (THC), has been linked to a variety of negative outcomes later in life [4–6], including increased subsequent consumption of “harder” drugs [7–12]. Given the posited role of the mesolimbic dopamine (DA) system as the final common pathway for addiction [13], it is not surprising that brain regions such as the ventral tegmental area (VTA), medial prefrontal cortex (mPFC), and nucleus accumbens (NAc) are critically important in mediating the long-lasting effects of adolescent THC on responses to psychoactive drugs later in life [10, 14–16]. Much like use of other psychoactive drugs [17–20], adolescent THC usage can lead to a hyperdopaminergic state [15] that then facilitates a variety of neuroadaptations that promote addiction. Despite this common framework, our mechanistic understanding of how adolescent THC causes persistent changes that linger until adulthood is limited.

Given that THC carries an addictive liability, it is critically important to understand the interactions between adolescent THC usage and exposure to addictive drugs later in life, for example prescription opioids. Adolescent THC use increases the likelihood of misusing addictive drugs by approximately 2–5x, with odds ratios between ~3.67 and 5 for opioids [21]. The long-hypothesized link between adolescent cannabis use and opioid misuse [22–24] is supported by the observation that cannabinoids and opioids may both activate the midbrain DA system, perhaps via common mechanisms of mu opioid receptor activation [25]. In addition, cross-sensitization of THC and morphine has been demonstrated [26], and adolescent THC exposure can alter expression of genes related to the endogenous opioid system [10], as well as potentiate opioid-seeking behaviors later in life [10, 27], providing further evidence that these drugs may work via common mechanisms. This is an important question because, despite the goal to reduce the clinical usage of opioids, opioids remain the primary treatment for post-operative pain management. In addition, approximately eighty percent of those who use heroin began using opioids for pain management [28, 29]. Therefore, it is important to understand the long-lasting impacts of THC use on the brain to mitigate the negative effects that adolescent THC may have on behavioral responses to opioids later in life.

In this study, we examine how adolescent THC exposure influences the behavioral and neural responses to morphine administration, withdrawal, and reinstatement during adulthood. We administered a moderate dose of THC (5 mg/kg) for 14 days starting at postnatal day 30 (PD30), during adolescence. After a washout period during which mice developed to adulthood, we then tested a variety of opioid-induced behaviors by administering morphine and assessing differential effects in those with or without a history of adolescent THC. After undergoing a forced abstinence period following repeated morphine injections, we also assessed how adolescent THC administration influences brain-wide activity patterns following a moderate dose of morphine, as well as how adolescent THC administration alters connectivity onto DA neurons in the VTA.

## Methods

All procedures were approved by the University of California, Irvine’s Institutional Animal Care and Use Committee (IACUC) and carried out in accordance with the National Institutes of Health guidelines for the care and use of animals. Adolescent male and female C57BL/6J mice were weaned and sexed at PD21 and group housed with same-sex littermates. A total of 40 male and 39 female mice were used for behavioral experiments, though not all mice were used for all experiments. Mice were housed in standard individually ventilated cages with corncob bedding and two cotton nestlets for enrichment. Lights were on a 12 hours on/12 hours off cycle (7:30–7:30) and rooms had controlled temperature (22 °C ± 2 °C) and humidity (55-65%). Mice received ad libitum access to food and water. Mice were housed in the Gillespie Neuroscience Research Facility (GNRF), except for during adolescent THC treatment. Mice were transferred to the McGaugh Hall mouse facility following weaning at approximately P25 where they received THC or vehicle injections, after which they were returned to the GNRF at approximately P45.

### Adolescent THC administration

Mice were handled and weighed daily. On PD30, mice began daily injections of either 5 mg/kg THC or vehicle, which was prepared daily by dissolving in 5% Tween80 in saline. After 14 days of daily injections (PD30-PD43), mice were left in their home cage undisturbed (aside from weekly cage changes and transfer back to GNRF) until PD70 when behavioral testing began.

### Drugs

THC was provided by the NIDA Drug Supply Program and Cayman Chemicals (Ann Arbor, MI) and administered at 5 mg/kg. Morphine was purchased from Patterson Veterinary Supply and was administered at 10 mg/kg for CPP and locomotor testing, and 5 mg/kg for reinstatement [30].

### Quantification and statistical analysis

Statistics for all studies were calculated using GraphPad Prism 10 software. All statistical parameters are reported in Supplemental Table 1. Statistical significance between direct comparisons was assessed by unpaired or paired t-tests. When multiple conditions were compared, one- or two-way ANOVAs were first performed, then t-tests were then performed with Tukey’s multiple comparisons corrections. In conditions where multiple comparisons corrections were performed and the results were still considered significant, asterisks were presented. Error bars represent s.e.m. throughout. For all figures, ns *P* > 0.05, *P ≤ 0.05, **P ≤ 0.01, ***P ≤ 0.001, ****P ≤ 0.0001.

Details of behavioral tests, cFos analysis, and RABV circuit mapping experiments can be found in the supplemental methods.

## Results

### Adolescent THC exposure history reduces morphine-induced locomotion, with no impact on morphine reward

**Fig. 1:**
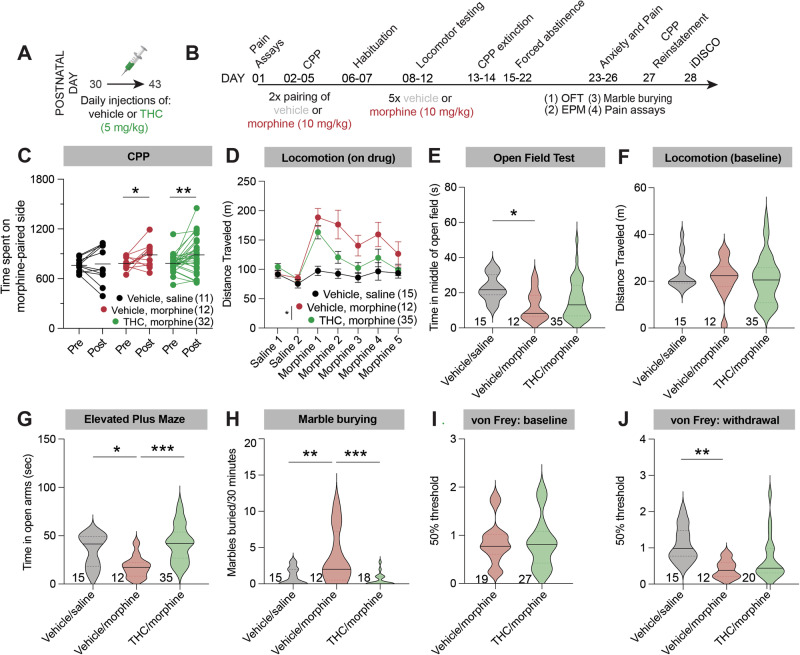
Effects of adolescent THC exposure on behavioral responses to morphine administration later in life. **A** Timeline of THC or vehicle injections during adolescence. Injections were repeated once per day for 14 total days. **B** Timeline of morphine injections and behavioral testing during adulthood. **C** Time spent in the morphine-paired chamber in the pre-test and post-test (in the vehicle/saline-paired group, both chambers were paired with saline). Vehicle/saline 762 s pre, 778 s post, *p* = 0.22; Vehicle/morphine 783 s pre vs. 887 s post, *p* = 0.038; THC/morphine 785 s pre vs. 887 s post, *p* = 0.0017. **D** Mice treated with THC during adolescence showed an overall reduction in morphine-induced locomotion relative to mice treated with saline during adolescence. Repeat measures two-way ANOVA between conditions *p* = 0.045; Tukey’s multiple comparisons tests morphine day 1 *p* = 0.68, morphine day 2 *p* = 0.24, morphine day 3 *p* = 0.31, morphine day 4 *p* = 0.50, morphine day 5 *p* = 0.75. Vehicle/saline-treated mice are shown for comparison but were not included in statistical tests. Two-way ANOVA only included morphine injection days, not habituation days. For this and all figures, error bars = SEM, ns *p* > 0.05, *p < 0.05, **p < 0.01, ***p < 0.001, ****p < 0.0001. **E** Repeated morphine administration led to a reduction in the time spent in the center of an open field relative to mice treated with saline during adolescence, and saline during adulthood. Adolescent THC exposure blunted this reduction. One-way ANOVA *p* = 0.027; pairwise t-tests, vehicle/saline vs. vehicle/morphine 22.66 s vs. 12.55 s, *p* = 0.030; vehicle/saline vs. THC/morphine 22.66 s vs. 15.85 s, *p* = 0.077; vehicle/morphine vs. THC/morphine 12.55 s vs. 15.85 s, *p* = 0.59. **F** There was no difference in locomotion during the five-minute open field test between groups. Two-way ANOVA *p* = 0.63. **G** Repeated morphine administration led to a reduction in the time spent in the open arms of an elevated plus maze, indicative of anxiety-like behavior relative to mice treated with saline during adolescence, and saline during adulthood. Adolescent THC exposure prevented this morphine-induced anxiety. One-way ANOVA *p* = 0.0004; pairwise t-tests, vehicle/saline vs. vehicle/morphine 34.97 s vs. 17.84 s, *p* = 0.035; vehicle/saline vs. THC/morphine 34.97 s vs. 42.39 s, *p* = 0.35; vehicle/morphine vs. THC/morphine 17.84 s vs. 42.39 s, *p* = 0.0002. **H** Repeated morphine administration led to an elevation in the number of marbles buried, indicative of anxiety-like behavior relative to mice treated with saline during adolescence, and saline during adulthood. Adolescent THC exposure prevented this morphine-induced anxiety. One-way ANOVA *p* = 0.0006; pairwise t-tests, vehicle/saline vs. vehicle/morphine 0.93 vs. 3.67, *p* = 0.0048; vehicle/saline vs. THC/morphine 0.93 vs. 0.50, *p* = 0.83; vehicle/morphine vs. THC/morphine 3.67 vs. 0.50, *p* = 0.0006. **I** Mice given saline vs. THC during adolescence showed no difference in baseline mechanical sensitivity. Vehicle/morphine vs. THC/morphine 0.85 g vs 0.86 g, *p* = 0.93. **J** Repeated morphine administration led to a reduction in the 50% mechanical threshold, indicative of pain hypersensitivity relative to mice treated with saline during adolescence, and saline during adulthood. Adolescent THC exposure blunted this morphine-induced pain hypersensitivity. One-way ANOVA *p* = 0.0032; pairwise t-tests, vehicle/saline vs. vehicle/morphine 1.09 vs. 0.42, *p* = 0.0027; vehicle/saline vs. THC/morphine 1.09 vs. 0.69, *p* = 0.052; vehicle/morphine vs. THC/morphine 0.42 vs. 0.69, *p* = 0.30.

We first exposed adolescent mice (PD30) to a daily 5 mg/kg/day dose of THC (or its vehicle) for 14 days (Fig. 1A). This is considered a moderate dose in mice that is comparable to doses self-administered by humans [31, 32], and 14 days of administration leads to long-lasting physiological and behavioral changes in mice and rats [4, 33–39]. Following vehicle or THC administration, mice were left in their home cages undisturbed besides weekly cage changes until postnatal day 70, at which time morphine administration commenced (Fig. 1B). To test how adolescent THC exposure history impacts morphine reward, we conducted a conditioned place preference (CPP) test using 10 mg/kg morphine. We induced a significant preference to the morphine-paired side in both vehicle and THC exposed animals, to an approximately equivalent extent (paired t-tests vehicle, saline *t*_10_ = 0.3108, *p* = 0.7623; vehicle, morphine *t*_11_ = 2.352, *p* = 0.0384; THC, morphine *t*_31_ = 3.136, *p* = 0.0037; Fig. 1C).

To determine whether adolescent THC treatment impacted morphine-induced locomotion, we measured locomotor behavior while mice were in an open field. Following a two-day habituation protocol where mice were given saline injections, 10 mg/kg morphine was given once per day for five days to test the sensitization or tolerance to morphine’s effects on locomotion. We observed that morphine increased locomotion the most following the first dose, and this elevation was reduced with each subsequent dose, for both groups (two-way ANOVA conditions term *F*_1,62_ = 6.776, *p* = 0.0115; Fig. 1D). We also found that adolescent THC exposure overall blunted morphine-induced locomotion during the five-day morphine administration window, though no individual day was significantly different between the groups following multiple comparisons corrections. These data indicate that adolescent THC exposure reduced morphine-induced locomotion following morphine exposure later in life.

### Adolescent THC exposure prevents the development of anxiety-related behavior and pain hypersensitivity during morphine withdrawal

We next assessed whether mice treated with THC during adolescence were differentially impacted by withdrawal from repeated morphine administration. Following the five days of morphine given for locomotor testing and two days of no morphine for CPP extinction, mice were left in their home cages for an additional 8 days, for a total abstinence period of ten days (Fig. 1B). Following this ten-day forced abstinence, mice were tested for anxiety-like behavior using three tests: the open field test, elevated plus maze, and marble burying tests. In the open field test, mice are both compelled to explore new environments, and avoid open areas; less time spent exploring the center of the arena is interpreted as anxiety-like behavior. We found that repeated morphine injections reduced time spent in the center of the open field, and THC history reduced this anxiety-like behavior (One-way ANOVA *F*_2,59_ = 3.838, *p* = 0.0271; multiple comparisons-corrected unpaired t-test vehicle/morphine vs. vehicle/saline 95% CI −19.40 to −0.8231, *p* = 0.0298; Fig. 1E). Notably, no differences were observed in overall locomotion during this five-minute test (One-way ANOVA *F*_2,59_ = 0.4621, *p* = 0.6322; Fig. 1F). The elevated plus maze tests how mice balance their tendency to explore against their preference for enclosed spaces, and reduction in the time spent exploring the open arms is interpreted as anxiety-like behavior. Mice treated with repeated morphine injections spent less time in the open arms of the elevated plus maze relative to those treated with repeated saline injections (One-way ANOVA *F*_2,59_ = 9.026, *p* = 0.0004; multiple comparisons-corrected unpaired t-test vehicle/morphine vs. vehicle/saline 95% CI −33.23 to −1.021, *p* = 0.0347; Fig. 1G). However, adolescent THC exposure blocked the reduction in time spent in the open arms induced by repeat morphine injection, suggesting that adolescent THC exposure suppressed withdrawal-induced anxiety behaviors in response to adult opioid exposures (multiple comparisons-corrected unpaired t-test THC/morphine vs. vehicle/morphine 95% CI 10.64 to 38.46, *p* = 0.0002; Fig. 1G). Lastly, in the marble burying test mice tend to bury marbles that are exposed in the bedding of a new cage, and more marbles buried is interpreted to reflect increased anxiety-like behavior. We found that mice undergoing forced abstinence following repeat morphine injections buried more marbles after the 30-min test, whereas adolescent THC administration reduced this number, consistent with a reduced anxiety-like behavior (one-way ANOVA *F*_2,42_ = 8.931, *p* = 0.0006; multiple comparisons-corrected unpaired t-tests vehicle/morphine vs. vehicle/saline 95% CI 0.7527 to 4.714, *p* = 0.0048; THC/morphine vs. vehicle/morphine 95% CI −5.073 to −1.261, *p* = 0.0006; Fig. 1H). Therefore, the results from each of these tests support the conclusion that adolescent THC exposure decreases the expression of anxiety-like behaviors in morphine-treated mice.

Next, we used a von Frey assay to examine the effects of adolescent THC exposure on mechanical hypersensitivity, both before and after morphine injection. In addition to an increase in anxiety-like behavior, mice undergoing physiological withdrawal following repeated opioid administration show an increased pain sensitivity [40, 41]. Mice treated with repeated THC doses during adolescence showed no difference in mechanical thresholds when tested one day prior to morphine CPP testing (unpaired t-test *t*_44_ = 0.08606, *p* = 0.9318; Fig. 1I). However, when tested again following repeat morphine exposure and forced abstinence, mice given repeated morphine showed an increased mechanical hypersensitivity relative to saline-treated controls, and adolescent THC treated reduced the extent of this hypersensitivity (one-way ANOVA *F*_2,44_ = 6.550, *p* = 0.0032; multiple comparisons-corrected unpaired t-tests vehicle/morphine vs. vehicle/saline 95% CI −1.143 to −0.2146, *p* = 0.0027; Fig. 1J; THC/morphine vs. vehicle/morphine 95% CI −0.1656 to 0.7097, *p* = 0.2973). Together, these results indicate that adolescent THC exposure reduces the expression of morphine withdrawal-induced behaviors following repeated morphine administration during adulthood.

### Adolescent THC exposure enhances drug-induced reinstatement of morphine CPP

**Fig. 2:**
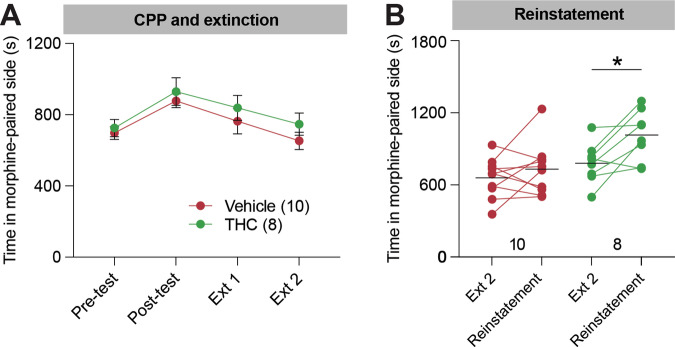
Mice treated with THC during adolescence show an elevation in drug-induced reinstatement of morphine-seeking behaviors later in life. **A** Time spent in the morphine-paired chamber during CPP pre-test, post-test, extinction, and reinstatement. Mice treated with THC or vehicle during adolescence showed a similar trajectory of CPP and extinction. **B** Quantification of the time spent in the morphine-paired chamber in the reinstatement task relative to the final extinction day. Vehicle/morphine-treated mice 660 s vs 732 s, *p* = 0.33, *n* = 10; THC/morphine-treated mice 781 s vs. 1015 s, *p* = 0.015, *n* = 8.

Following forced abstinence, we next assessed whether mice differentially reinstated CPP following a 5 mg/kg exposure of morphine in the CPP box. Drug exposure following abstinence can reinstate previously extinguished drug-associated behaviors such as CPP, and therefore can serve as a model of relapse in mice. Both groups of morphine-treated mice showed normal CPP (e.g., Fig. 1C). Following CPP, mice then underwent extinction (Fig. 2A). While mice treated with vehicle during adolescence did not significantly reinstate CPP following the priming 5 mg/kg morphine injection using this protocol, mice treated with THC during adolescence did significantly reinstate their CPP (paired t-tests vehicle/morphine *t*_9_ = 1.039, *p* = 0.3260; THC/morphine *t*_7_ = 3.212, *p* = 0.0148; Fig. 2B). This means that adolescent THC exposure facilitates reinstatement of CPP in response to a moderate priming morphine dose during adulthood.

### Whole-brain analysis shows differences in brain activity following drug-primed reinstatement in adolescent vehicle- vs. THC-treated mice

**Fig. 3:**
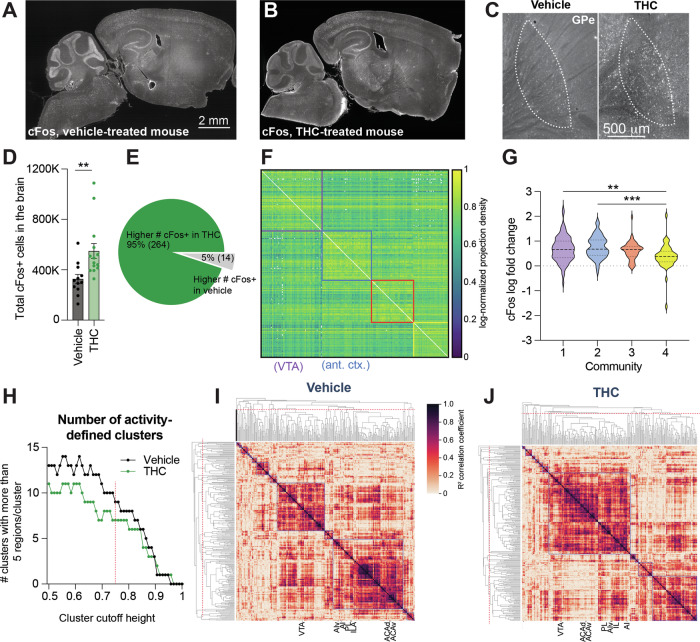
Brain-wide activity patterns in adolescent vehicle- and THC-treated mice following a reinstatement dose of morphine. **A** Representative sagittal section of a cFos-stained brain of an animal treated with vehicle during adolescence and following a reinstatement dose of morphine. Scale, 2 mm. **B** Representative section of a cFos-stained brain of an animal treated with THC during adolescence and following a reinstatement dose of morphine. **C** Representative sagittal images of the GPe in adolescent vehicle-treated vs. THC-treated mice. Scale, 500 μm. **D** More cFos-expressing cells were detected in adolescent THC-treated mice than vehicle-treated mice following a dose of 5 mg/kg morphine (averages of 548,481 vs. 325,789 cells, *p* = 0.0051). **E** Pie chart showing the percentage of brain regions with elevated or depressed cFos-expressing cells in adolescent THC-treated mice relative to adolescent vehicle-treated mice. **F** Anatomical connectivity matrix of the mouse brain, broken into four communities based on the Louvain method for community detection. **G** Log fold-change in number of cFos labeled cells between THC and Vehicle for all regions grouped by network community (One-way ANOVA *p* = 0.0002; purple (1) vs. blue (2) 0.68 vs. 0.75, *p* = 0.76; purple (1) vs. red (3) 0.68 vs. 0.61, *p* = 0.82; purple (1) vs. yellow (4) 0.68 vs. 0.38, *p* = 0.0024; blue (2) vs. red (3) 0.75 vs. 0.61, *p* = 0.31; blue (2) vs. yellow (4) 0.68 vs. 0.38, *p* = 0.0002; red (3) vs. yellow (4) 0.61 vs. 0.38, *p* = 0.05). **H** The number of hierarchical clusters in adolescent vehicle-treated and THC-treated mice across a range of heights for cluster cut-offs in hierarchical trees. The THC-treated mice show reduced modularity across a range of cutoff values. The dotted red line in this and (**I**, **J**) equals the cutoff used for cluster definition in (**I**, **J**) (0.75). **I** Hierarchical clustering of regions based on the cFos correlation matrix in adolescent vehicle-treated mice. The VTA is within a separate activity-defined cluster from the frontal cortex regions of interest (prelimbic cortex, PL; infralimbic cortex, ILA; anterior insula cortex, AI; anterior insula cortex, ventral part, AIv; ACAd, anterior cingulate cortex, dorsal part; ACAv, anterior cingulate cortex, ventral part). **J** Hierarchical clustering of regions based on the cFos correlation matrix in adolescent THC-treated mice. The VTA co-clusters with regions of the anterior cortex in these mice.

Given that adolescent THC-treated mice showed morphine-induced reinstatement whereas vehicle-treated controls did not, we next assessed what brain regions may be responsible for this effect. To do this, we perfused the mice 60 min following the reinstatement test, cleared the brains using iDISCO + , and immunostained for the immediate early gene cFos to obtain a brain-wide map of recent neuronal activity (Fig. 3A–C). We observed an overall elevation of recent neuronal activity in response to 5 mg/kg morphine in mice treated with THC during adolescence than vehicle, as evidenced by a higher number of cFos+ cells upon morphine exposure (unpaired t-test vehicle/morphine vs. THC/morphine *t*_24_ = 3.079, *p* = 0.0051; Fig. 3D, E). This increase was exhibited in most brain regions, while only a small number of brain regions showed a reduction in cFos labeling in adolescent THC-treated mice, which included both the medial and lateral habenula (Supplemental Figs. 1–3, Supplemental Table 2). We counted cells manually to test the accuracy of cells detected by ClearMap, and found significant correlation between manual counts and ClearMap counts across four representative regions: the globus pallidus externus (GPe), prelimbic area, hippocampus, and midline group of the dorsal thalamus (MTN) (Supplemental Table 3–4, Supplemental Fig. 5). These regions tended to show differences in cFos staining and were expected to show different trends of over or undercounting of cFos labeling by ClearMap. The averaged ratio of the number of ClearMap counts to the number of manual counts was 0.843 for the GPe, 1.12 for the prelimbic area, 1.14 for the hippocampus, and 1.18 for the MTN. Linear regression models were fit on ClearMap counts as an independent variable and manual counts as a dependent variable, with R^2 values of 0.86 for GPe, 0.78 for prelimbic area, 0.98 for hippocampus, and 0.91 for MTN. Slope coefficients for the models all were relatively close to each other at 0.976 ± 0.0371 for hippocampus, 0.766 ± 0.0623 for MTN, 1.054 ± 0.109 for GPe, and 0.920 ± 0.125 for the prelimbic area. These regression results imply that trends in cFos labeling detected by ClearMap reflect trends in the actual cFos labeling.

As the total cFos labeling was increased in the brains of mice treated with THC, we wanted to assess whether this elevation was larger for some pathways or whether the activation was uniform across the brain. To test this, we built a quantitative connectivity matrix of the mouse brain, using the Allen Mouse Brain Connectivity Atlas as a template [42]. Doing so revealed the existence of four major network communities within the brain, largely consisting of subcortical regions including the VTA (community 1, purple), cortical regions (community 2, blue), the hippocampus and related connections (community 3, red), and mid/hindbrain regions (community 4, yellow) (Fig. 3F). We then assessed the relative increase in cFos labeling of regions in each community in adolescent THC- vs. vehicle-treated mice following a reinstatement opioid dose. We found a significant increase in the log fold-change of cFos-labeled cells in THC vs vehicle-treated mice in the purple and blue communities relative to the yellow community, suggesting that the subcortical and cortical modules both showed an overall larger increase in recent activity relative to mid/hindbrain regions in THC-treated mice (One-way ANOVA *F*_3,254_ = 6.638, *p* = 0.0002; multiple comparisons-corrected unpaired t-tests purple vs. yellow 95% CI 0.08193 to 0.5109, *p* = 0.0024; blue vs. yellow 95% CI 0.1467 vs. 0.5937, *p* = 0.0002; Fig. 3G).

Following this analysis, we wanted to assess if these community-specific changes in cFos activation were accompanied by any changes in the functional relationships of different brain regions. To quantitatively assess functional modularity, we built a brain-wide functional correlation matrix for adolescent THC-treated and vehicle-treated mice based on their cFos labeling (Figs. 3H–J, S4). We performed hierarchical clustering on both correlation matrices across a range of parameters. Across a wide parameter range, adolescent THC-treated mice exhibited fewer recent activity-defined clusters than vehicle-treated mice, reflecting an overall reduced modularity in brains from adolescent THC-treated mice (Fig. 3H). We then compared a specific clustering in this range to see how various structures and known circuits are organized. We focused specifically on the VTA and frontal cortex regions, given their interconnectivity and known contributions to substance misuse [43–46]. While in vehicle-treated mice the VTA and frontal cortex regions separated into different clusters, reflecting their separation into different networks (Fig. 3I), in THC-treated mice the IL, PL, ACC, and AI all co-clustered with the VTA, indicating a higher level of correlated activity amongst these regions in adolescent THC-treated mice (Fig. 3J). These findings overall show that the increased activation of cortical and subcortical pathways in adolescent THC-treated vs vehicle-treated mice after reinstatement is accompanied by a collapse in modularity of pathways in adolescent THC-treated mice, including a tighter clustering of the VTA with frontal cortex regions.

### Adolescent THC exposure changes connectivity to ventral tegmental area dopamine cells

**Fig. 4:**
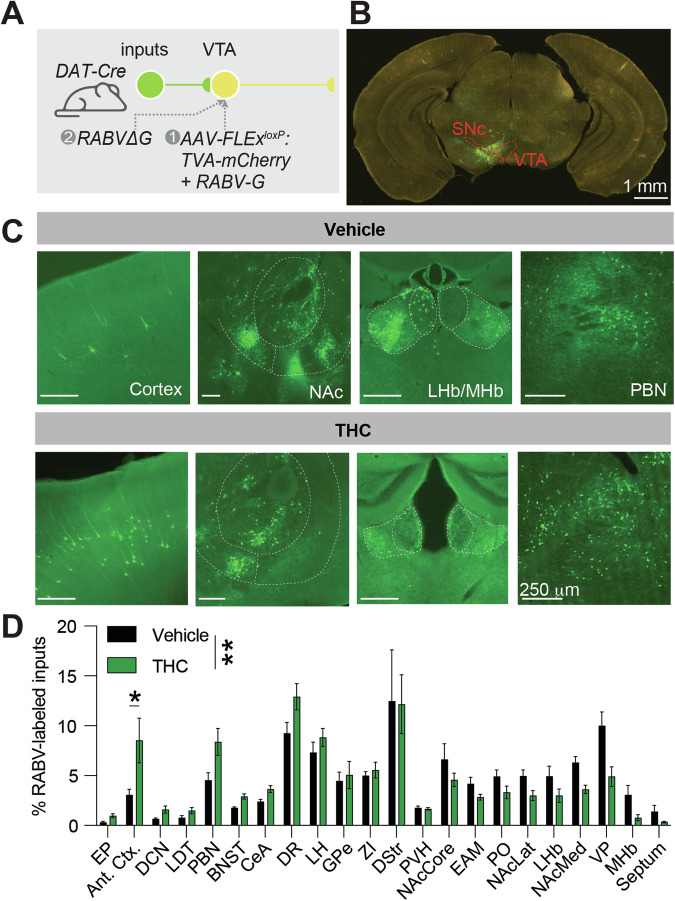
Adolescent THC exposure changes input connectivity to VTA^DA^ cells. **A** Schematic of experiments. **B** Representative image of the ventral midbrain of a DAT-Cre mouse showing starter cells in the VTA and medial SNc. Scale, 1 mm. **C** Representative images of input cell populations in several brain sites, including the anterior cortex, NAcMed, NAcCore, NAcLat, MHb, LHb, and PBN. Scale, 250 μm. **D** Bar graph plot showing the percentage of RABV-labeled inputs in control vs. THC-treated mice. Two-way ANOVA, interaction *p* = 0.0062. Only the anterior cortex was significantly different following correction for multiple comparisons (corrected *p*-value *p* = 0.029).

The VTA is a critical brain region for the development of a variety of drug-induced behavioral adaptations, including drug reward, sensitization, and withdrawal. To test whether adolescent THC exposure modified the input control to VTA^DA^ cells, we mapped inputs to these cells using the rabies virus (RABV) monosynaptic input mapping method [19, 47]. RABV-labeled input cells were observed throughout the brain, as in previous studies (Fig. 4A–C) [19, 47]. Overall, the input labeling pattern was significantly different between vehicle-treated and THC-treated mice (2-way ANOVA interaction *F*_21,176_ = 2.058, *p* = 0.0062; Fig. 4D). While several brain regions showed a visible difference in connectivity, for example elevated connectivity from cortical regions comprising the anterior cortex (infralimbic cortex, prelimbic cortex, anterior insula cortex, anterior cingulate cortex, and the anterior motor cortex) [19, 43, 47, 48], parabrachial nucleus (PBN) and deep cerebellar nuclei (DCN), only the anterior cortex was significantly different following correction for multiple comparisons (multiple comparisons-corrected unpaired t-test 95% CI 0.2923 to 10.66, corrected *p*-value *p* = 0.029).

**Fig. 5:**
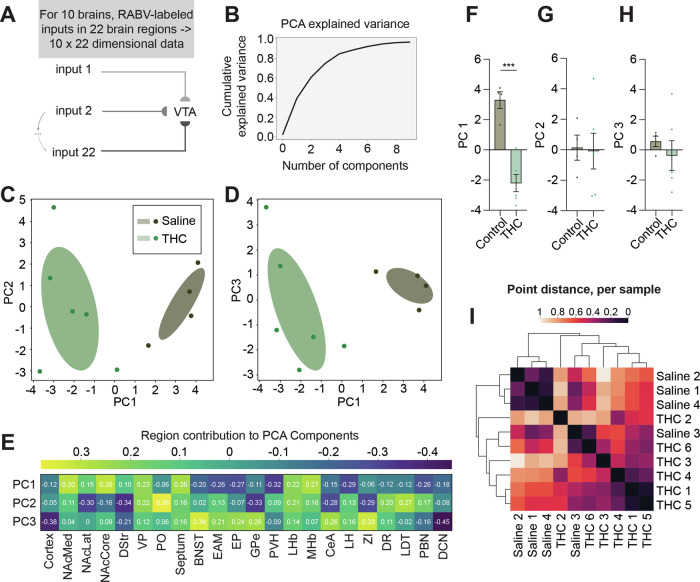
Identification of inputs driving the difference in connectivity to VTA^DA^ cells following adolescent THC exposure. **A** Schematic and explanation for how PCA analysis was carried out on the RABV tracing dataset. **B** Proportion of cumulative variance explained by each principal component. **C** Plot of PC1 and PC2 from brains of control and THC-treated mice. **D** Plot of PC1 and PC3 from brains of control and THC-treated mice. **E** Heatmap of the contributions of each brain region, or feature, in the data to PCs 1 through 3. Comparison of total PC values for each brain along (**F**) PC 1, (**G**) PC 2, and (**H**) PC 3. **F** Control 3.3 vs. THC −2.2, *p* = 0.0002. **G** Control 0.13 vs. THC −0.09, *p* = 0.89. **H** Control 0.57 vs. THC −0.38, *p* = 0.48. **I** To further quantify relationships between brains and conditions, the Euclidean distance between each brain from control and THC-treated mice in the PC1 versus PC2 coordinate space was calculated. These distances were then plotted in a heatmap with brains being organized by similarity assessed by hierarchical clustering.

This traditional approach to assessing differences in connectivity is statistically conservative, and only assesses potential differences in connectivity, one input at a time. To reveal further patterns in the data, we have recently employed a dimensionality reduction approach that enables us to detect larger-scale differences in input patterns between brains of mice undergoing a variety of treatments [47, 49]. We therefore first used this approach to assess which inputs best differentiated THC-treated vs. control mice using principal component analysis (PCA). We found that 3 PCs were sufficient to explain approximately 75% of the variance in the data, and thus we focused only on these 3 PCs (Fig. 5A, B). We found that PC1 completely separated THC-treated and control mice, whereas PC2 and PC3 did not (Fig. 5C–D). Control mice had high PC1 values, which were driven predominately by strong contributions from nucleus accumbens medial shell (NAcMed), core (NAcCore), ventral pallidum (VP), septum, lateral habenula (LHb) and medial habenula (MHb), while THC-treated mice had low PC1 values, which were driven by the bed nucleus of the stria terminalis (BNST), extended amygdala area (EAM), entopeduncular nucleus (EP), paraventricular nucleus of the hypothalamus (PVH), lateral hypothalamus (LH), and PBN (Fig. 5E). To quantify these differences, we plotted each brain’s location on each PC, and performed t-tests. Differences between THC-treated and control groups were strongly significant along PC1 (unpaired t-test *t*_8_ = 6.559, *p* = 0.0002), and not different along PCs 2 and 3, as expected (unpaired t-test PC2 *t*_8_ = 0.1407, *p* = 0.8916; unpaired t-test PC3 *t*_8_ = 0.7424, *p* = 0.4791; Fig. 5F–H). To further quantify relationships between brains and conditions, the Euclidean distance between each brain in the PC1 versus PC2 coordinate space was calculated, and the relationships plotted as a correlogram. THC-treated and control brains separated largely by condition, as expected (Fig. 5I).

## Discussion

Here, we tested the effects of adolescent THC exposure on later-life morphine-induced behaviors, brain-wide activity following morphine reinstatement, and midbrain DA cell synaptic connectivity. We found that adolescent THC exposure blunts morphine-induced locomotion and withdrawal anxiety/pain hypersensitivity and enhances morphine-primed CPP reinstatement. This broad range of effects is consistent with findings from studies that have explored the influence of THC on morphine-induced behavioral changes [10, 14, 26, 27, 50]. Here, we extend these previous studies by linking brain-wide activity patterns following CPP reinstatement and show that adolescent THC exposure creates long-lasting changes in input connectivity to VTA^DA^ cells, principally from the anterior cortex. Our study thus adds to a rich literature on the subject by implicating several discrete brain regions as potential drivers of the long-lasting effects of adolescent THC exposure on behavioral and brain responses to opioids later in life.

### Adolescent THC exposure promotes a behavioral response to opioids akin to those observed in adolescence

It is perhaps surprising that THC exposure blunts morphine withdrawal-induced anxiety across several behavioral assays, as well as pain sensitivity. Interestingly, the behavioral profile is consistent with the behavioral responses observed following exposure to drugs during adolescence [51–55], where adolescent mice experience potentiated drug reward with only mild withdrawal symptoms relative to adult mice receiving equivalent drug exposures. This effect could be mediated by an occlusion of opioid-induced epigenetic changes by those elicited following adolescent THC exposure. Such an effect was previously observed following juvenile exposure to oxycodone; in this case, oxycodone induced long-lasting epigenetic changes, for example an elevation of the repressive histone modification H3K27me3 on genes that control DA transmission in the VTA [56]. Therefore, one hypothesis is that adolescent THC exposure may result in a similar imbalance of reward/aversion as is seen in responses to drugs during adolescence by occluding compensatory responses to drug exposure that typically occur in adulthood, but not adolescence. This could result in, for example, withdrawal from repeated opioid injection failing to induce a compensatory induction in anxiety-related behaviors and pain hypersensitivity. In this case, reversal of these epigenetic marks may restore mice to basal levels of susceptibility. Future studies are required to identify the potential cellular loci of these effects, as well as what the epigenetic consequences of early life THC exposure may be.

### Whole-brain cFos analysis indicates enhanced engagement of mesocorticolimbic circuits following adolescent THC exposure

In addition, we found that adolescent THC administration induces higher levels of drug-primed reinstatement, consistent with previous observations assessing drug- or stress-induced reinstatement [8–10]. Here, we extend these observations by linking these results to brain-wide cFos activation in adolescent vehicle- vs. THC-treated mice. We found that adolescent THC-treated mice had overall higher numbers of cFos+ cells in response to a 5 mg/kg dose of morphine than adolescent vehicle-treated controls. In addition, we found that adolescent THC-treated mice have higher levels of correlated activity in the mPFC and AI with the VTA than adolescent vehicle-treated mice, indicating enhanced functional connectivity between these regions (Fig. 3I, J). We previously showed that activation of these cortical inputs to the VTA promotes reinforcement through increasing DA release in the NAc [43]. Therefore, the elevated functional connectivity that we observe between these regions in adolescent THC-treated mice may promote greater levels of DA release in the NAc than in adolescent vehicle-treated mice and is one mechanism by which adolescent THC-treated mice may more strongly reinstate morphine CPP following a priming dose of morphine.

However, one limitation of cFos-based analyses is that they do not consider anatomical connections. Viral methods such as the monosynaptic RABV circuit mapping technique can specifically label synaptically-connected cells [57, 58]. Furthermore, we recently showed that the extent of input labeling is dependent on the activity of input connections [19, 20, 59]. Therefore, our RABV mapping experiments have the unique ability to explore the changes in connectivity to VTA^DA^ cells following repeated THC exposure, without a preconceived bias as to which inputs may be altered. We have previously used this assay to define how drugs change inputs to VTA^DA^ cells, enabling us to identify several pathway-specific changes that have important consequences for the development of drug-induced behavioral adaptations [47]. Here, the most prominent difference between mice given adolescent THC exposure vs. vehicle is that THC-treated mice show a pronounced increase in inputs from the anterior cortex (Fig. 4D). Heightened activation of this connection is consistent with our cFos results and would be expected to be rewarding through elevating activity in VTA^DA^ cells. This hypothesis is consistent with the hyper-dopaminergic state of THC-treated animals previously reported [15], and the more robust response in these animals to a reinstatement dose of morphine. In addition, we observed an overall reduced modularity in adolescent THC-mice relative to adolescent vehicle-treated controls (Fig. 3H). A reduction in brain modularity is seen following use and withdrawal from addictive drugs [60–65] as well as brain disorders such as traumatic brain injury and Alzheimer’s-related dementia [66–71]. How this reduced modularity may influence behavioral responses to drugs, however, remains largely unknown.

### Adolescent THC exposure increases connectivity between the cortex and VTA^DA^ cells

In our study, we also tested the hypothesis that adolescent THC exposure alters connectivity onto VTA^DA^ cells. This hypothesis was supported by the observation that rats treated with THC during adolescence develop a hyper-dopaminergic state [15]; one potential mechanism that could mediate this effect would be a change in connectivity to facilitate an increase in DA release in downstream brain regions. Here, we found an elevation in inputs from the anterior cortex to VTA^DA^ cells, which we previously showed promotes reinstatement by facilitating DA release in the NAc [43]. Notably, we previously have shown that a single injection of a variety of drugs can cause long-lasting changes in input connectivity onto VTA^DA^ cells [19, 20, 59], with this signature being relatively similar regardless of the drug administered [19, 47], suggesting that these drugs induce similar changes in connectivity. The anterior cortex, particularly the mPFC, is thought to play important roles in the later stages of substance use disorder [45, 72–74]; inputs are reduced following a single injection of a “hard” drug [19, 47]. The reduction in connectivity from the anterior cortex following a single dose, and increase in connectivity following repeated doses of THC seen here, is thus consistent with that hypothesis of altered mPFC engagement during the addiction cycle. One additional point of note is that we previously showed that drugs increase connectivity from the GPe to the ventral midbrain, and that these changes are important for drug-induced behavioral adaptations including reward and sensitization [19, 20]. However, THC treatment did not induce a notable change in GPe inputs onto VTA^DA^ cells (Fig. 4D). Thus, while THC triggers several long-lasting changes in connectivity to VTA^DA^ cells, these appear to be distinct from those initiated by psychostimulants, nicotine, or morphine. Notably, our experiments utilized experimenter-administered morphine and THC, rather than self-administration, meaning that we are not capturing the volitional aspect of drug administration. Therefore, much remains to be explored about the interactions between THC and “hard drugs”, and further experiments will be needed to resolve these questions.

## Supplementary information

Supplemental Methods

Supplemental Table 1

Supplemental Table 2

Supplemental Table 3

Supplemental Table 4

## Data Availability

The datasets generated during and/or analyzed during the current study are available from the corresponding author on reasonable request.
