# Supplementary material for: Neural basis of adolescent THC-induced potentiation of opioid responses later in life
Source: Neuropsychopharmacology. 2024 Dec 10;50(5):818–27. doi: 10.1038/s41386-024-02033-8 (PMC11914220; doi:10.1038/s41386-024-02033-8)
Supplement: Supplementary file 1 — Supplemental Methods [file 41386_2024_2033_MOESM1_ESM.docx]

**METHODS:**

Behavioral Testing:

Assessing sex differences: We compared behavioral results for male and female mice for all tests. We did not observe any clear THC-related sex differences in any behavior; the only notable trend we observed was that morphine treatment (in the adolescent vehicle-treated group) induced a bigger reduction in the time spent in the center of the open field, however this effect was not observable in the THC-treated group. We therefore collapsed male and female data for all statistical tests reported herein.

Locomotion: The distance traveled in the open field was assessed during 30-minute recording sessions. Locomotion was assessed once per day for seven days, the first two following saline injections for habituation days, and the last five following injection of 10 mg/kg morphine.

Conditioned place preference: Custom two chamber acrylic boxes separated by a small corridor with distinct visual contexts on the walls of each side were used for CPP tests. On the pre-test day, mice were placed in the box with no barriers and were allowed to freely explore both sides for 30 minutes, and the time spent in each chamber was recorded. Following the pre-test, mice underwent two saline pairings and two morphine pairings. In the morning, the mice were given saline and paired with the preferred chamber, and in the afternoon, injected with morphine and paired with the non-preferred chamber. Control mice received saline in both contexts. On the post-test day, mice were again placed in the CPP chamber with no barriers, and the amount of time spent in each chamber was recorded. Data were plotted as the relative time spent on the drug-paired chamber in the post-test relative to the pre-test. Mice that spent over 1200 seconds on one side during the pre-test were excluded from analysis. A total of 4 mice from the vehicle/saline, 3 mice from the vehicle/morphine, and 3 mice from the THC/morphine groups were excluded based on these criteria.

For extinction, mice were placed back in the CPP chambers for 30 minutes over 2 sessions, with both sides accessible. No morphine or saline was given. For CPP reinstatement, mice were given a dose of 5 mg/kg morphine and placed into the CPP chamber with both sides accessible. Mice were excluded if they spent more time in the drug-paired chamber following extinction than before extinction. 2 mice were excluded from the vehicle/morphine group, and 2 mice were excluded from the THC/morphine group based on these criteria. Note that not all animals that were tested for CPP were tested for extinction and reinstatement, and not all animals were tested for anxiety-related behaviors and and pain sensitivity.

Elevated plus maze: Mice were placed at the end of one of the open arms, facing outwards. Mice were then able to freely explore the maze for 5 minutes, and the time spent in the open arms was recorded. Tests were performed in dim lighting ~25 lux.

Open field test: Mice were placed in a 40cm x 40cm square arena made of opaque plexiglass and were able to freely explore during a 5-minute test. During analysis, the arena was divided into nine equal square zones. The time spent in the center square for each mouse was calculated. Tests were performed under bright lighting conditions of ~1000 lux.

Marble burying: Mice were placed in a rat cage with 20 marbles placed on top of corn-cob bedding. After 30 minutes, the number of marbles that were over half-buried by each mouse was recorded.

Von Frey: The von Frey test was used to assess mechanical sensitivity. Mice were placed on top of a wire mesh platform, restrained underneath a glass beaker, and allowed to acclimate for at least 30 minutes. The mouse's right hindpaw was gently touched with different filaments, starting with the 0.4g filament. If the mouse showed a withdrawal response (such as lifting, shaking, or licking the paw), the experimenter stepped down to a lighter filament, and if the mouse did not respond, the experimenter stepped up to a thicker filament until a response occurred. The process continued with filaments of higher or lower forces for four additional turns following the first change in response. This up/down method was used to determine the mechanical force required to elicit a paw withdrawal response (50% threshold)^1^.

cFos induction and immunohistochemistry: One hour following injection of 5 mg/kg morphine (a dose used only for morphine-induced reinstatement), mice were perfused with PBS followed by 4% formaldehyde, and brains isolated and post-fixed overnight in 4% formaldehyde. The following day, brain clearing began using iDISCO^2^. The monoclonal rat anti-cFos antibody (Synaptic Systems lot #226017) in 5% NDS/PBS-Tween 0.1% was used at a 1:5000 dilution, with an incubation time of five days. Donkey anti-rat Cy3 (Jackson Immunoresearch) was then used at a dilution of 1:250. Brains were then cleared in dibenzyl ether and imaged using a Zeiss Z1 light-sheet microscope. ClearMap^3^ was then used to align the imaged brains to the Allen Brain Reference Atlas, and to quantify the number of cFos+ neurons in anatomically-defined brain regions. ClearMap parameters for cell detection were tuned individually for each brain on 100x100x100 pixel subsections in order to maximize the number of correctly labeled cells and minimize the number of incorrectly labeled cells (Supplemental Tables 3 and 4). 312 regions from the atlas were included in the ClearMap analysis, according to a default region schema. Layer-specific counts were not included in order to focus on mesoscale circuits. Ventricular systems and fiber tracts were excluded from analysis as well as these are often artifacts. Cells were also labeled manually in 100x100x100 pixel subsections centered around the globus pallidus externus (GPe), prelimbic area, hippocampus, or midline group of the dorsal thalamus (MTN). In cases where subsections had over 200 cells, the subsections were made 50x50x50 to ensure close attention to detail in counting. Linear regression models were fit relating the number of ClearMap-detected cells against the number of manually-detected cells for each of these regions separately.

Network Analysis: In order to relate functional connectivity using cFos labeling to anatomical connectivity, anatomical projection data from the Allen Mouse Brain Connectivity Atlas was used to construct a connectivity matrix among the brain regions in our analysis^4^. This atlas quantifies axonal arborization from AAV injections to measure projections from one region to another. We processed this data into a connectivity matrix similar as has been done in the literature with slight modification^5^. The density of each projection, or the sum or projecting pixels divided by the total pixels in that region, was normalized by dividing by the volume of injection, so as not to bias experiments with more virus injection than others. In a modification to the previous approaches, this quantity was also multiplied by the number of voxels in the injection structure to avoid overrepresentation of smaller regions. This quantity was computed for every pair of source and target regions to build a connectivity matrix. Projection densities of 0 were excluded such that there were no edges with weights of 0. We log-normalized these quantities to yield a more normal distribution and behave better in network analysis. Previously, hierarchical clustering has been used to find modules of regions that are more connected with each other. We followed applications in human brain functional networks^6^ and *C. elegans*^7^ and used the Louvain algorithm for community detection, which is more optimized for finding communities in large graphs. The Louvain algorithm was computed with a resolution parameter of 1, using the scit-kit network library^8^. This resolution parameter was selected as it led to the detection of communities with dozens of regions per community, yielding a mesoscale organization of the brain. The clusters were visualized in a heatmap showing the connectivity matrix of all regions, ordered first by community membership and second by their order in our ClearMap spreadsheets. Once the clusters were determined, the cFos log fold change between THC and Vehicle of their regions were compared using a one-way ANOVA followed by pairwise t-tests. Log fold change was used to reflect changes in cFos activation in regions of different sizes, as a difference quantification would cause regions with a smaller number of cells total that still have more active cells in THC to look the same as a region with a large number of total cells but very little change in THC.

Functional Analysis: Regional cFos counts across each mouse in the adolescent vehicle-treated and THC-treated conditions were dimensionally reduced using UMAP, so that regions with similar counts across the mice would be embedded close to each other^9^. Regions were clustered using HDBSCAN, which can identify hierarchical clusters in continuous data based on variations in density of the data^10^. Hierarchical clustering was also performed on the correlations between regions, to look more closely at their functional relationships. R^2^ value from Pearson correlations were computed for each pair of regions, based on their counts across mice of a given condition. Hierarchical clusterings were computed on correlation matrices using the average linkages methodology. For visualizations, the hierarchical clustering tree was cut at a height of 0.75 to determine the clusters (Figure 4I-J). The trees were cut for heights between 0.5 and 1 to observe trends in modularity across the two conditions. The seaborn library was used for all hierarchical clustering computations^11^.

Rabies virus circuit mapping: Rabies virus (RABV) monosynaptic tracing studies were carried out as previously described^12,13^, with minor modifications. On Day 0, 100 nL of a 1:1 volume mixture of AAV-CAG-FLEx^loxP^-TC and AAV-CAG-FLEx^loxP^-G was injected into the VTA of PD56 mice that had undergone two weeks of THC injections between PD30 and PD43. Fourteen days later, RABV was injected into the VTA. After recovery, mice were housed in a BSL2 facility for 5 days to allow RABV spread and GFP expression. Mice were then perfused with PBS followed by 4% formaldehyde and postfixed overnight. Fixed brains were cryoprotected by incubation in a 30% sucrose solution overnight, and frozen in OCT. Brains were then serially sliced on a cryostat into 60 um sections. GFP+ cells throughout the brain in 22 anatomically-defined input sites were counted manually, as previously performed^12,13^.

Viruses and injection coordinates: Coordinates used for the VTA were (relative to Bregma, midline, or dorsal brain surface and in mm): AP − 3.20, ML 0.4, DV − 4.2.

The titers of viruses, based on quantitative PCR analysis, were as follows:

AAV-CAG-FLEx^loxP^-TC, serotype 5, 2.4 x 10^13^ genome copies (gc)/ml

AAV-CAG-FLEx^loxP^-G, serotype 8, 1.0 x 10^12^ gc/ml

For RABV, the titer was performed on HEK 293T-TVA800 cells.

RABV (EnvA-pseudotyped, GFP-expressing), 5.0 x 10^8^ colony forming units (cfu)/ml

After scaling RABV data, PCA was applied so features were brain regions and observations were individual brains of mice treated with various drugs. Differences between mouse brains were visualized by plotting PC1, PC2, and PC3 (Figure 5J-L, Q-R). Ellipsoids for each condition were calculated and overlayed onto plots to visualize trends in PC values by condition. To further quantify relationships between brains and conditions, the Euclidean distance between each point (or each brain) in the PC1 versus PC2 coordinate space was calculated. These distances were then plotted in a heatmap with brains being organized by similarity assessed by hierarchical clustering (Figure 5M). Alternatively, for a summary by condition, these distances were averaged based on drug administered, and the resulting heatmap and clustering were made based on averaged Euclidean distance values (Figure 5S). Heatmaps and clustering were made using the clustermap function in the Python package seaborn with default parameters^11^. The RABV data that included single injections of psychostimulants, morphine, or nicotine, or two anesthetic doses of ketamine/xylazine, were presented in our published work^14^ and used here for sake of comparison. For these experiments, a single dose of 15 mg/kg cocaine, 1 mg/kg amphetamine, 2 mg/kg methamphetamine, 0.5 mg/kg nicotine, or 10 mg/kg morphine were given to adult mice, and RABV tracing was performed the following day. These mice were anesthetized with isoflurane-based anesthesia, as used in this manuscript. A separate group was anesthetized with a ketamine/xylazine mixture prior to both helper virus injection and RABV injection, and not given another drug. RABV was introduced during the second ketamine/xylazine injection.

Code and Data Availability: All code and data used in the study are available upon request. cFos samples used for the linear regression analysis in Supplemental Table 4 and Supplemental Figure 5 are available on zenodo at https://doi.org/10.5281/zenodo.14190694.

**
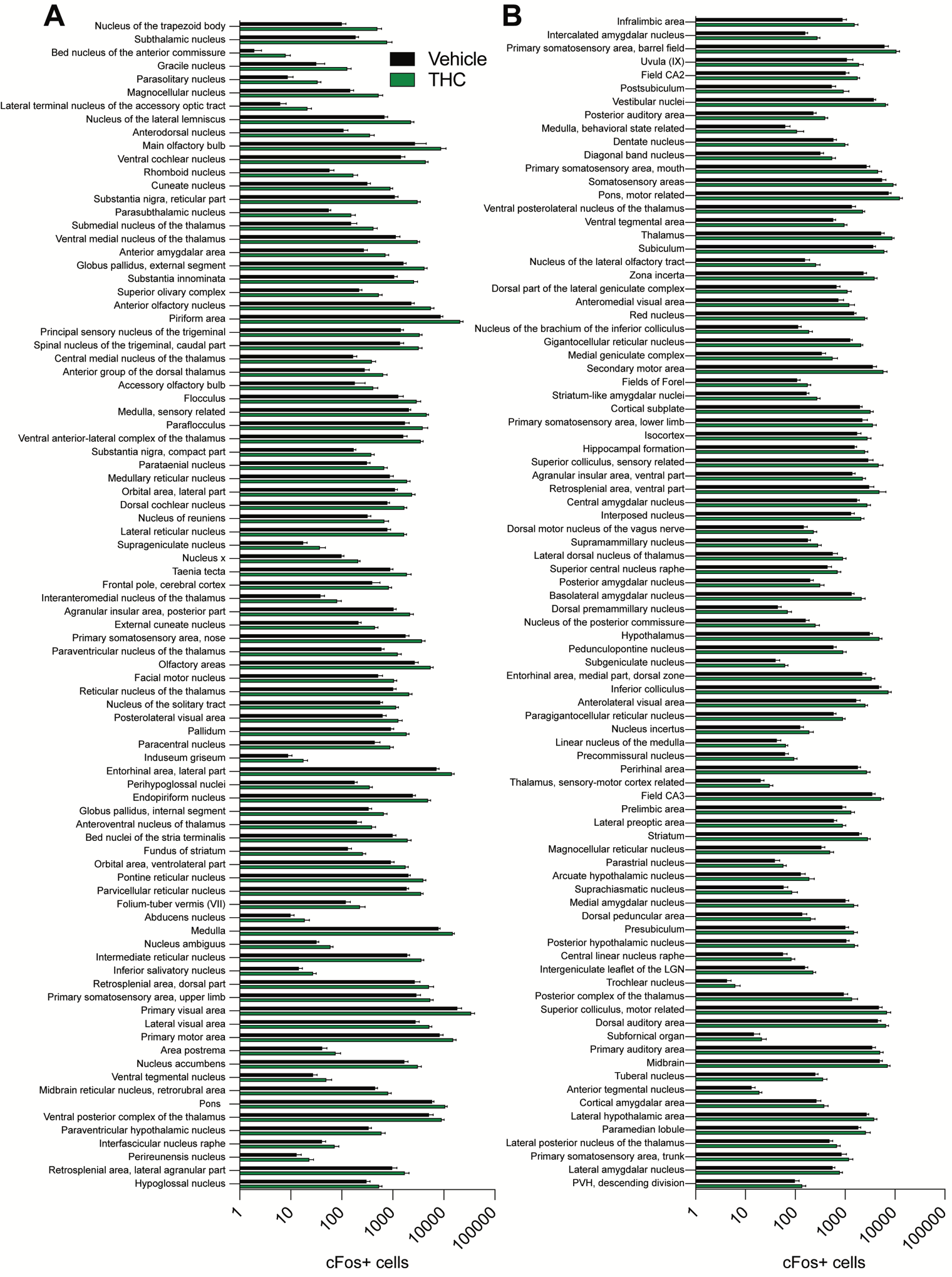
FIGURES:**

**Figure S1: Comparison of the number of cFos+ cells in each brain region following drug-primed reinstatement with 5 mg/kg morphine, in mice injected with vehicle or THC during adolescence.** Shown are regions with elevations in cFos labeling in mice receiving THC over those receiving vehicle, in order by the magnitude of difference between conditions. The rest of the data are plotted in Figure S2.

**
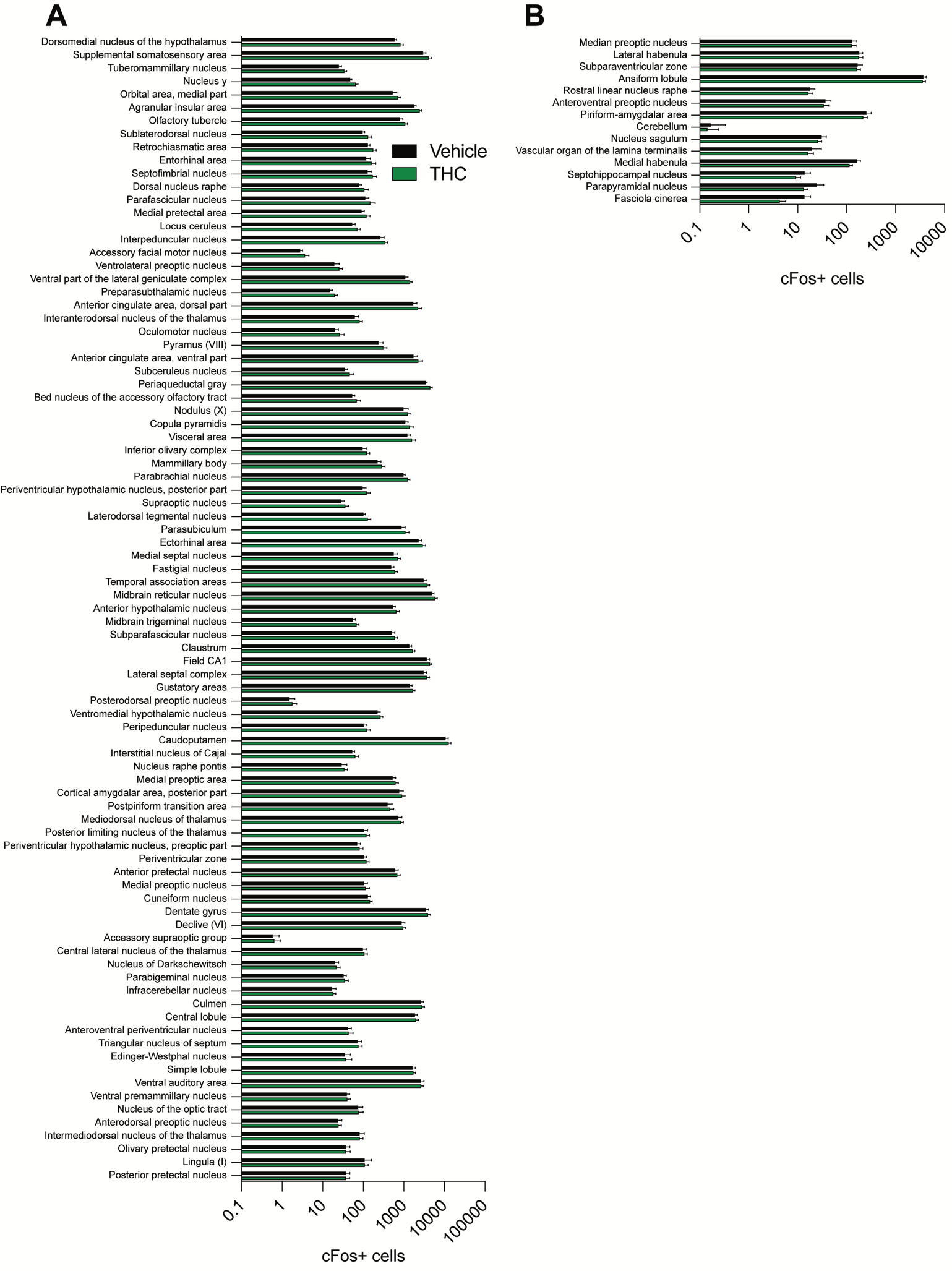
Figure S2: Continuation of Figure S1.** Shown are regions with **A** elevations in cFos labeling in mice receiving THC over those receiving vehicle, or **B** vehicle over those receiving THC.

**
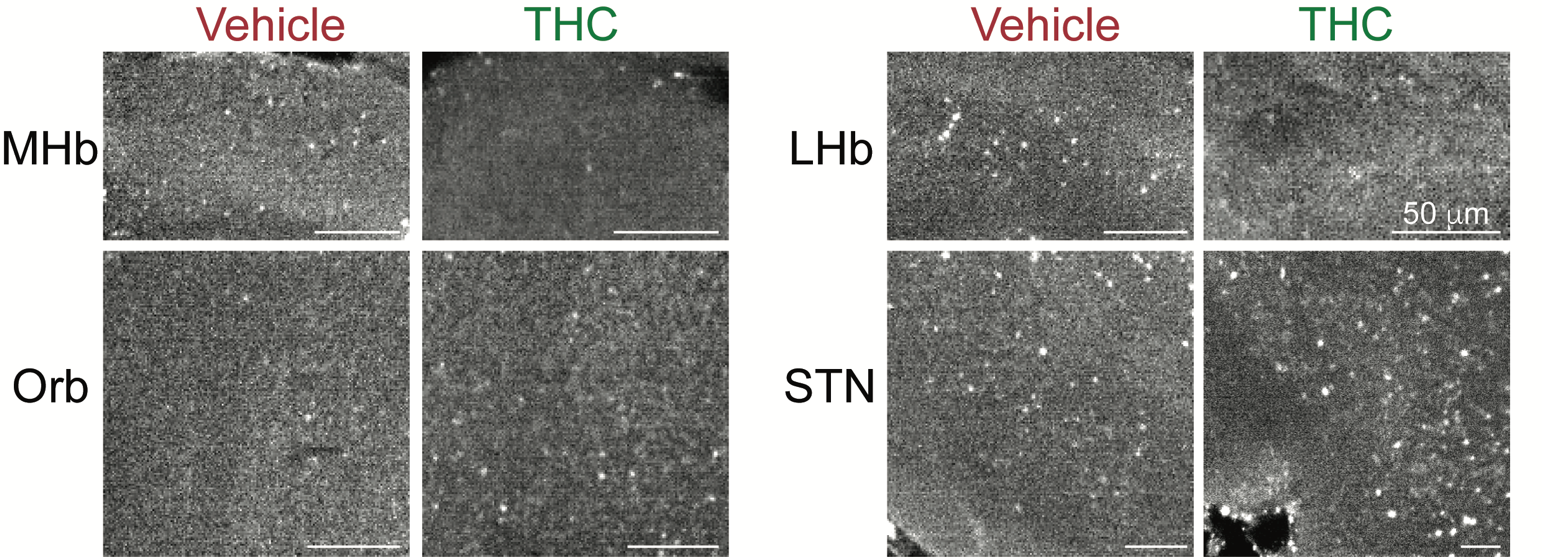
**

**Figure S3: Additional raw images of cFos-stained brains from mice treated with vehicle or THC during adolescence.** Shown are images from the MHb, LHb, orbitofrontal cortex (Orb), and subthalamic nucleus (STN).

**
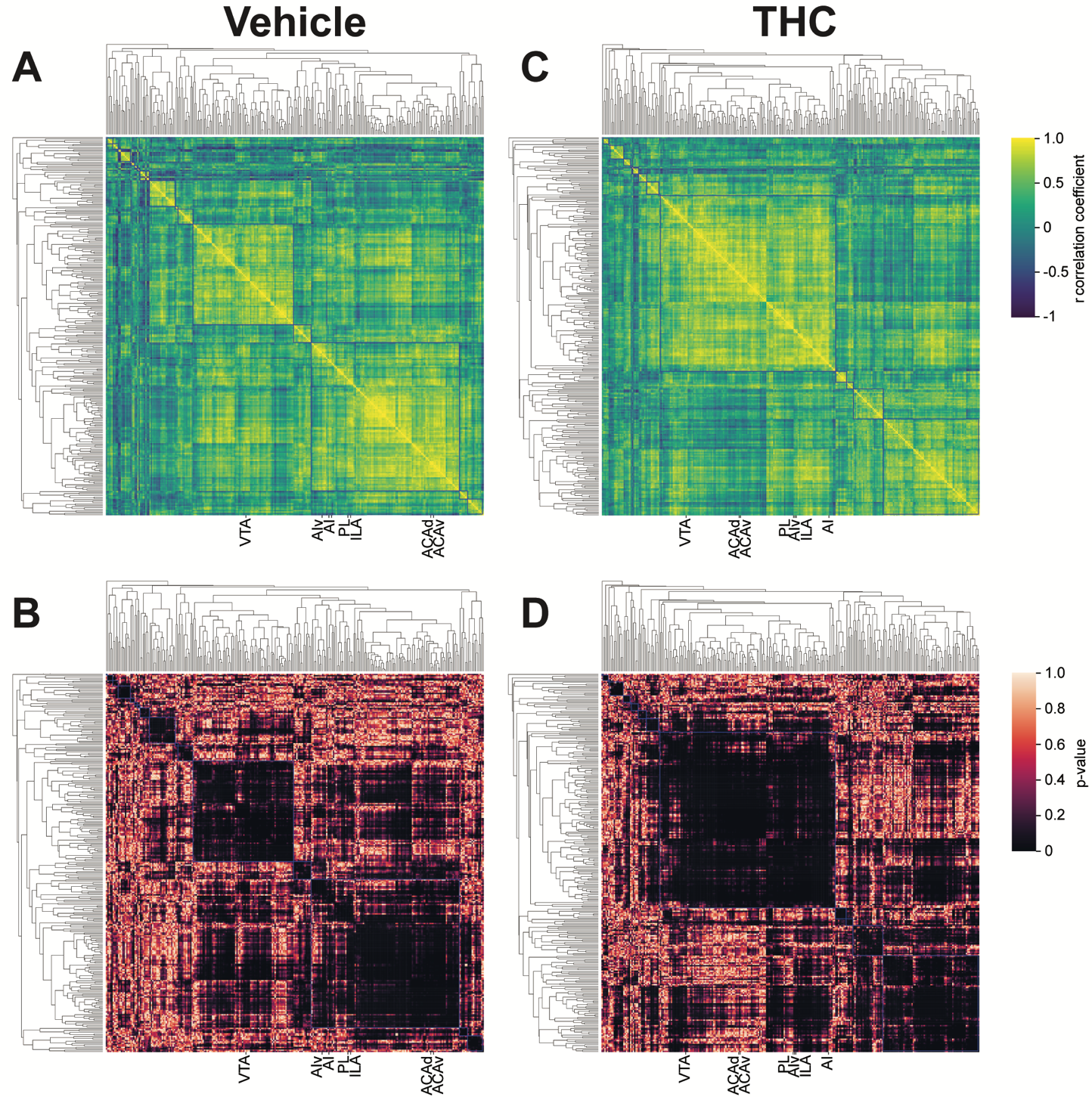
Figure S4: Correlograms for cFos data, showing either the r value or p-value for each set of brain regions in mice treated with vehicle or THC during adolescence, related to Figure 3I-J.** Vehicle-treated mice are shown in A-B, and THC-treated mice in C-D.

**
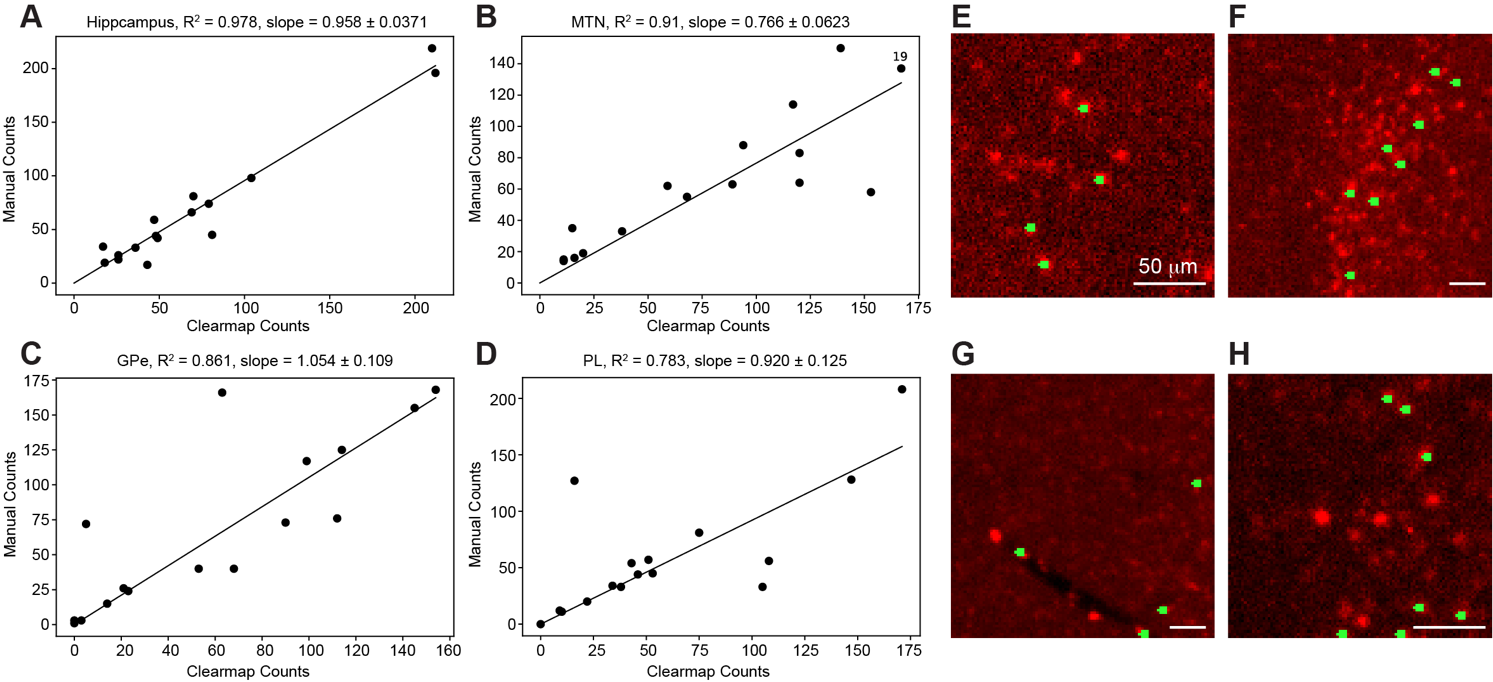
**

**Figure S5: Linear regression models relating ClearMap-derived counts of cFos cells to cells manually counted, related to Figure 3.** Shown are counts for the hippocampus (**A**)**,** midline group of the dorsal thalamus (MTN; **B**), GPe (**C**), and prelimbic area (PL; **D**)**.** Sample images are included as well showing the location of cells marked manually for the hippocampus (**E**), MTN (**F**), GPe (**G**), and PL (**H**). Note that these slices come from 3D image stacks in which each cell is marked only on a single frame as is specified by ClearMap, so some cells that are apparent on these images may have been marked on different frames**.**

**Supplemental Table 1: Statistics table for all statistical comparisons in this manuscript.**

**Supplemental Table 2: Raw data for cFos quantifications, related to data shown in Figure 3 and Figures S1-5.**

**Supplemental Table 3: Parameters for ClearMap cFos cell detection on all brains, related to data shown in Figure 3 and Figures S1-5.**

**Supplemental Table 4: Manual and ClearMap-derived counts of cFos cells in samples images used for linear regression analysis, related to data shown in Figure 3 and Figures S1-5.**

**SUPPLEMENTAL REFERENCES**:

1. Chaplan, S. R., Bach, F. W., Pogrel, J. W., Chung, J. M. & Yaksh, T. L. Quantitative assessment of tactile allodynia in the rat paw. *J Neurosci Methods* **53**, 55–63 (1994).

2. Renier, N. *et al.* IDISCO: A simple, rapid method to immunolabel large tissue samples for volume imaging. *Cell* **159**, 896–910 (2014).

3. Renier, N. *et al.* Mapping of Brain Activity by Automated Volume Analysis of Immediate Early Genes. *Cell* **165**, 1789–1802 (2016).

4. Oh, S. W. *et al.* A mesoscale connectome of the mouse brain. *Nature* **508**, 207–214 (2014).

5. Harris, J. A. *et al.* Hierarchical organization of cortical and thalamic connectivity. *Nature* **575**, 195–202 (2019).

6. Meunier, D., Lambiotte, R., Fornito, A., Ersche, K. D. & Bullmore, E. T. Hierarchical modularity in human brain functional networks. *Front Neuroinform* **3**, (2009).

7. Bassett, D. S. *et al.* Efficient physical embedding of topologically complex information processing networks in brains and computer circuits. *PLoS Comput Biol* **6**, (2010).

8. Muller, B., Richards, A. J., Jin, B. & Lu, X. GOGrapher: A Python library for GO graph representation and analysis. *BMC Res Notes* **2**, (2009).

9. McInnes, L., Healy, J. & Melville, J. UMAP: Uniform Manifold Approximation and Projection for Dimension Reduction. *ArXiv* **1802.03426**, 1–51 (2018).

10. McInnes, L., Healy, J. & Astels, S. hdbscan: Hierarchical density based clustering. *The Journal of Open Source Software* **2**, 205 (2017).

11. Waskom, M. Seaborn: Statistical Data Visualization. *J Open Source Softw* **6**, 3021 (2021).

12. Beier, K. T. *et al.* Circuit Architecture of VTA Dopamine Neurons Revealed by Systematic Input-Output Mapping. *Cell* **162**, 622–634 (2015).

13. Beier, K. T. *et al.* Rabies screen reveals GPe control of cocaine-triggered plasticity. *Nature* **549**, 345–350 (2017).

14. Bartas, K. *et al.* Drug-induced changes in connectivity to midbrain dopamine cells revealed by rabies monosynaptic tracing. *Elife* **13**, RP93664 (2024).
